# Early clinical outcomes of Portico and Edwards Sapien 3 valve prosthesis in transcatheter aortic valve replacement: propensity-matched analysis

**DOI:** 10.3389/fcvm.2024.1400626

**Published:** 2024-07-15

**Authors:** Uwe Primessnig, Julia M. Wiedenhofer, Tobias D. Trippel, Carina M. Loddenkemper, Helene Schrader, Anna Brand, Sebastian Spethmann, Karl Stangl, Arash Haghikia, Ulf Landmesser, Leif-Hendrik Boldt, Florian Blaschke, Gerhard Hindricks, Simon H. Sündermann, Herko Grubitzsch, Volkmar Falk, Henryk Dreger, Mohammad Sherif

**Affiliations:** ^1^ Department of Cardiology, Angiology and Intensive Care Medicine, Deutsches Herzzentrum der Charité, Campus Virchow Klinikum, Berlin, Germany; ^2^ Corporate Member of Freie Universität Berlin and Humboldt-Universität zu Berlin, Charité—Universitätsmedizin Berlin, Berlin, Germany; ^3^ DZHK (German Centre for Cardiovascular Research), Berlin, Germany; ^4^ Department of Cardiology, Angiology and Intensive Care Medicine, Deutsches Herzzentrum der Charité, Campus Charité Mitte, Berlin, Germany; ^5^ Department of Cardiology, Angiology and Intensive Care Medicine, Deutsches Herzzentrum der Charité, Campus Benjamin Franklin, Berlin, Germany; ^6^ Department of Cardiothoracic and Vascular Surgery, Deutsches Herzzentrum der Charité, Berlin, Germany

**Keywords:** transcatheter aortic valve replacement (TAVR), surgical aortic valve replacement (SAVR), self-expandable portico valve prosthesis, balloon-expandable Edwards Sapien 3 valve prothesis, permanent pacemaker (PPM) implantation

## Abstract

**Introduction:**

There is a lack of real-world data directly comparing different valve prostheses for transaortic valve replacement (TAVR). We aimed to compare early clinical outcomes at 30-days between the self-expandable Portico valve (Abbott) with the balloon-expandable Edwards Sapien 3 valve (Edwards Lifesciences) (ES3).

**Methods:**

Out of 1,901 patients undergoing TAVR between January 2018 and December 2021, all patients who received either Portico valve or ES3 valve via transfemoral TAVR were matched using nearest-neighbor (1:1) propensity scoring. Primary endpoints were single safety endpoints and early safety composite endpoints defined by Valve Academic Research Consortium-2 (VARC-2) criteria. The secondary endpoint was to analyze risk predictors for new permanent pacemaker (PPM) implantation in TAVR.

**Results:**

Out of 661 complete cases, a total of 434 patients were successfully matched based on age, sex, Euro Score II and STS-score. In the matched cohort, 217 received either a Portico or valve and 217 received an ES3 valve. The VARC-2 early safety composite scores indicated a significantly greater overall 30-day safety risk in the Portico group at 9.2% (*n* = 20) compared to 3.7% (*n* = 8) in the ES3 group (*p* = 0.032). The requirement for new permanent pacemaker (PPM) implantation was also higher in the Portico group, at 21.2% (*n* = 46) vs. 13.4% (*n* = 29) in the ES3 group (*p* = 0.042). 30-day mortality was higher was 3.7% (*n* = 8) in Portico group compared to 0.9% in ES3 group (*p* = 0.11). Furthermore, implantation of the Portico valve was identified as a significant risk predictor for new PPM implantation, alongside higher age, preprocedural atrioventricular block (AVB) and longer total procedure duration.

**Conclusion:**

This study shows significantly higher rates of early clinical complications for Portico valve prostheses compared to ES3. These findings should be especially taken into consideration when selecting valve prosthesis for high-risk patients.

## Introduction

Transcatheter aortic valve replacement (TAVR) has evolved as a widely accepted treatment modality not only for patients with severe aortic stenosis (AS) who are at high or extreme surgical risk but increasingly for those at intermediate and even low risk (1–3). Although significant advancements in TAVR have been made, early generation devices have certain limitations. These limitations include the inability to retrieve or reposition the valve after full expansion, potential hemodynamic compromise during implantation and the requirement for large access sheath sizes (4). Moreover, self-expanding valves are associated with a higher incidence of new permanent pacemaker (PPM) implantation. However, the precise impact is unclear (5, 6). New-generation TAVR devices have been developed to overcome limitations and reduce complications associated with first-generation devices (6). The choice between balloon-expandable and self-expandable valve prostheses is typically made on an individual basis considering the patient's characteristics, the anatomy of the aortic valve (e.g., size, shape, calcification) and the presence of calcification or tortuosity of access vessels, evaluated by the professional expertise of the institutional heart team. The Portico valve (Abbott), introduced in 2012, is a self-expandable prosthesis with large, open cells within a nitinol stent frame and bovine leaflets positioned intra-annular. A re-sheathable design allows repositioning during intervention (7). The Edwards Sapien 3 (ES3; Edwards Lifesciences) valve, is a balloon-expandable third-generation valve prosthesis incorporating a lower profile to minimize vascular complications. It features a polyethylene terephthalate outer skirt aimed at reducing PVL and the necessity for post-dilation. Additionally, the valve's low frame design with an open cell geometry allows unimpeded access to the coronary arteries (8, 9). This study aims to investigate and report real-world data on early clinical outcomes of patients who underwent TAVR using either the self-expandable Portico valve or the new generation ES3 valve at a high-volume center.

## Methods

In this retrospective comparative-cohort study, a total of 1,901 patients, who underwent TAVR between January 2018 and December 2021 at Charité University Medical Center were included. Complete case analysis was performed for baseline characteristics, endpoints, and matching variables, excluding cases with missing data for these variables. For variables with missing data below the threshold of 1%, missing values were not imputed or completed, and the available cases were analyzed accordingly. Patients who received valves other than the study valves or underwent non-transfemoral TAVR, were also excluded from further analysis. To ensure a balanced comparison, Portico valve recipients were matched to an equal number of ES3 valve recipients using propensity score matching. TAVR procedures were performed based on the institutional heart team's collaborative decision, following comprehensive evaluation including either computed tomography or transesophageal echocardiography. Valve prosthesis selection was based on the decision of the heart team or operator. Clinical outcomes were defined in accordance with the Valve Academic Research Consortium-2 (VARC-2) consensus document and assessed during a 30-day follow-up period. The primary endpoints were all-cause mortality, bleeding, vascular complications, stroke, acute kidney injury, new PPM implantation and new atrioventricular block. Furthermore, the study evaluated the VARC-2 early safety composite endpoint, which consisted of all-cause mortality, all stroke (disabling and non-disabling), life-threatening bleeding, acute kidney injury (AKIN stage 2 or 3), coronary obstruction requiring repeat intervention, major vascular complications, and valve-related dysfunction requiring repeat procedures. The secondary objective of this study was to identify risk predictors for new PPM implantation. Clinical data was extracted retrospectively from electronic medical records. All analyses were performed on anonymized datasets to protect patient privacy and confidentiality. The study was conducted following the STROBE (Strengthening the Reporting of Observational Studies in Epidemiology) checklist to ensure comprehensive and transparent reporting and had ethical approval from the Charité's ethics committee (EA4/131/23).

## Statistical analysis

Propensity scores were estimated using a logistic regression model based on age, sex, Euro Score II and STS-Score. Using the propensity scores, a 1:1 nearest neighbor matching was conducted between the Portico group and the ES3 group. We used a caliper width of 0.25 standard deviations of the logit of the propensity score to match patients. Comparative analysis between groups for continuous variables was performed using the Student's *t*-test or the Wilcoxon-Mann-Whitney *U*-test, while Chi-square test was used for categorical variables. Normality was assessed using Shapiro-Wilk test. Continuous variables are expressed as means (± standard deviations) or medians [interquartile ranges] (IQR). Categorical variables are presented as absolute numbers and percentages. Multivariable logistic regression analysis was performed to identify risk predictors for new in-hospital PPM implantation after TAVR adapting for a total of 19 potential confounders. All statistical analyses were conducted using R 4.2.3 (10).

## Results

**Figure 1:**
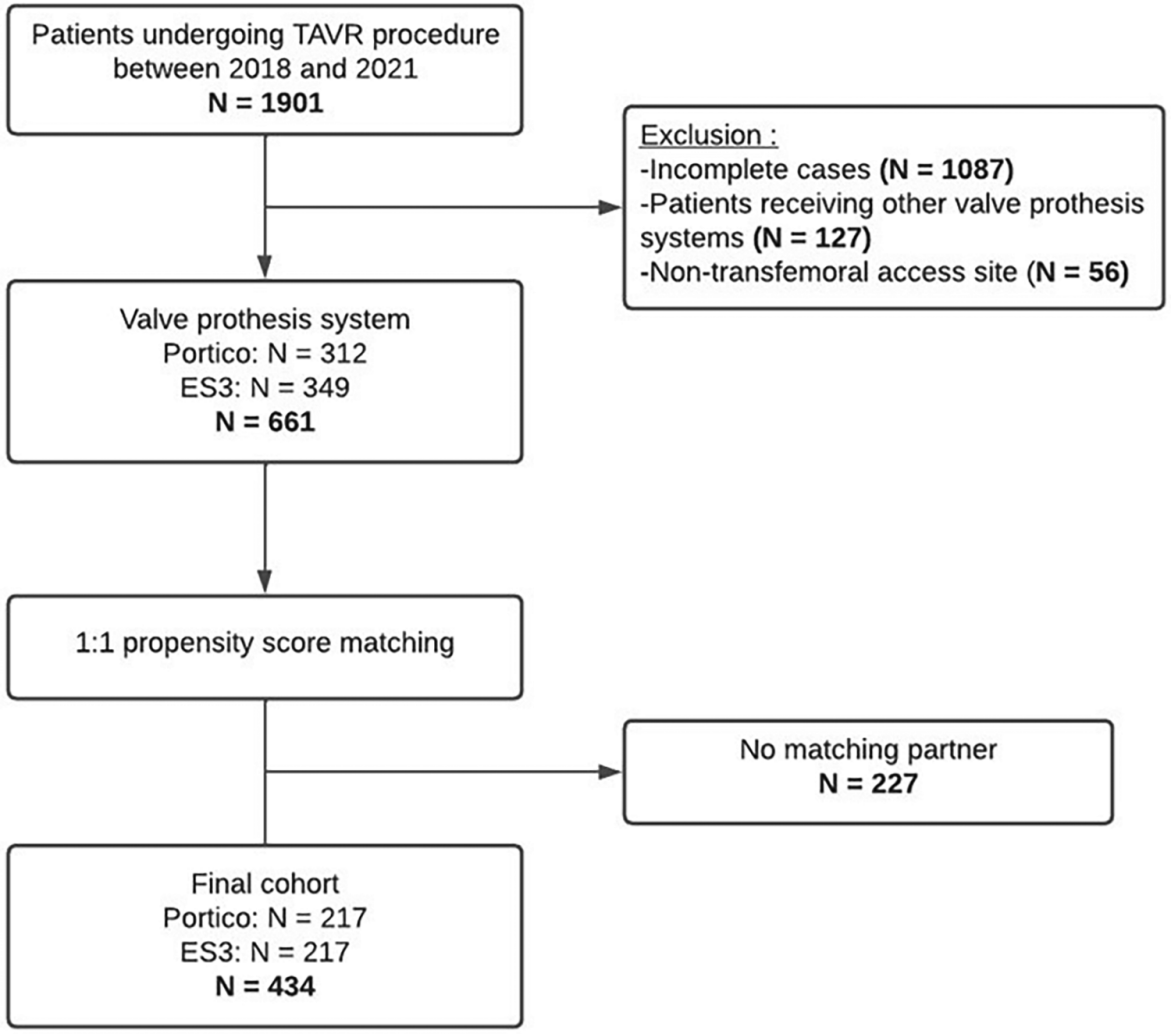
Study population selection.

Among the initial cohort of 1,901 patients who underwent TAVR between January 2018 and December 2021, a total of 1,087 incomplete cases were excluded from the study. After excluding these cases, the remaining cohort consisted of 814 patients. However, within this group, 153 patients were excluded additionally, as they did not meet inclusion criteria due to receiving different types of valve prostheses or undergoing TAVR with other than transfemoral access site chosen. Out of 661 patients in the final cohort, a total of 434 were matched. Among the matched cohort 217 patients received either the Portico valve and 217 patients the ES3 valve. The study population selection is displayed in Figure 1.

### Baseline characteristics

Table 1 presents the baseline characteristics of the two groups, demonstrating a well-balanced distribution. There were no significant differences between the Portico and ES3 groups in terms of demographic characteristics, comorbidities and pathological preprocedural rhythms. The baseline left ventricular ejection fraction (LVEF) was 54.6 (11.1) % in the Portico group compared to 52.0 (12.3) % in the ES3 group, showing a statistically significant difference (*p* = 0.03). However, no significant differences were observed in other baseline echocardiographic parameters, including the mean aortic valve gradient [41.1 (14.4) mmHg vs. 39.9 (14.5) mmHg; *p* = 0.499] and peak aortic velocity [4.42 (4.29) m/s vs. 4.14 (3.14) m/s; *p* = 0.382] for the Portico and ES3 groups, respectively.

**Table 1:** Preprocedural baseline characteristics of unmatched and matched study cohort.

		Unmatched			Matched		
		Portico	Edwards Sapien 3	*P*-value	Portico	Edwards Sapien 3	*P*-value
		*N* = 312	*N* = 349		*N* = 217	*N* = 217	
Female		194 (62.2%)	111 (31.8%)	<0.001**	100 (46.1%)	88 (40.6%)	0.287
Age (years)		82.5 (5.20)	78.8 (6.58)	<0.001**	81.1 (5.17)	80.0 (6.50)	0.143
BMI (kg/m^2^)		27.2 (5.12)	27.6 (5.62)	0.463	27.7 (5.36)	27.5 (5.54)	0.564
Obesity (BMI ≥30)		82 (26.3%)	88 (25.2%)	0.823	51 (23.5%)	63 (29.0%)	0.23
Hyperlipoproteinemia		187 (59.9%)	183 (52.4%)	0.063	114 (52.5%)	131 (60.4%)	0.121
Arterial hypertension		277 (88.8%)	310 (88.8%)	1	192 (88.5%)	190 (87.6%)	0.882
Diabetes mellitus		95 (30.4%)	113 (32.4%)	0.653	78 (35.9%)	72 (33.2%)	0.614
Previous coronary heart disease		195 (62.5%)	209 (59.9%)	0.543	134 (61.8%)	128 (59.0%)	0.624
History of atrial fibrillation		129 (62.5%)	139 (39.8%)	0.751	82 (37.8%)	87 (40.1%)	0.694
History of renal insufficiency		123 (39.4%)	114 (32.7%)	0.084	88 (40.6%)	70 (32.3%)	0.09
NYHA	I	1 (0.3%)	11 (3.2%)	0.019*	1 (0.5%)	5 (1.8%)	0.075
	II	66 (21.2%)	93 (26.6%)		44 (20.3%)	58 (26.7%)	
	III	220 (70.5%)	212 (60.7%)	0.019*	158 (72.8%)	136 (62.7%)	0.075
	IV	25 (8.0%)	33 (9.5%)		14 (6.5%)	19 (8.8%)	
Euro Score II		4.30 [0.670, 44.7]	3.53 [0.930, 43.0]	0.001*	3.85 [0.670, 44.7]	4.18 [0.930, 43.0]	0.513
STS-Score		4.25 [0.998, 29.5]	3.07 [0.790, 26.1]	<0.001**	3.60 [0.998, 19.6]	3.32 [0.894, 26.1]	0.363
Preprocedural rhythm	Atrial fibrillation	72 (24.5%)	65 (19.3%)	0.138	45 (22.3%)	42 (20.0%)	0.656
	Atrioventricular block	37 (12.6%)	65 (19.3%)	0.028*	26 (12.9%)	41 (19.8%)	0.082
	Brunch bundle block	79 (27.1%)	99 (29.7%)	0.515	58 (29.0%)	62 (30.0%)	0.919
LVEF (%)		54.9 (10.7)	53 (11.9)	0.063	54.6 (11.1)	52.0 (12.3)	0.03*
Aortic valve area (cm^2^)		0.751 (0.158)	0.782 (0.190)	0.114	0.774 (0.156)	0.775 (0.200)	0.416
Aortic valve mean gradient (mmHG)		40.8 (14.5)	40.0 (14.9)	0.875	41.1 (14.4)	39.9 (14.5)	0.499
Aortic valve peak gradient (mmHG)		64.9 (22.3)	62.3 (23.1)	0.347	64.3 (22.7)	61.6 (22.4)	0.245
AV Vmax (m/s)		4.23 (3.59)	4.05 (2.52)	0.857	4.42 (4.29)	4.14 (3.14)	0.382

Values are displayed as frequencies (percent), mean (standard deviation) and median [interquartile range]. AV Vmax, peak aortic jet velocity; BMI, body mass index; LVEF, left ventricular ejection fraction; NYHA, New York Heart Association Classification of Heart Failure.

* Significant *p* < .05.

** Highly significant *p* < .001.

### Procedural details

**Figure 2:**
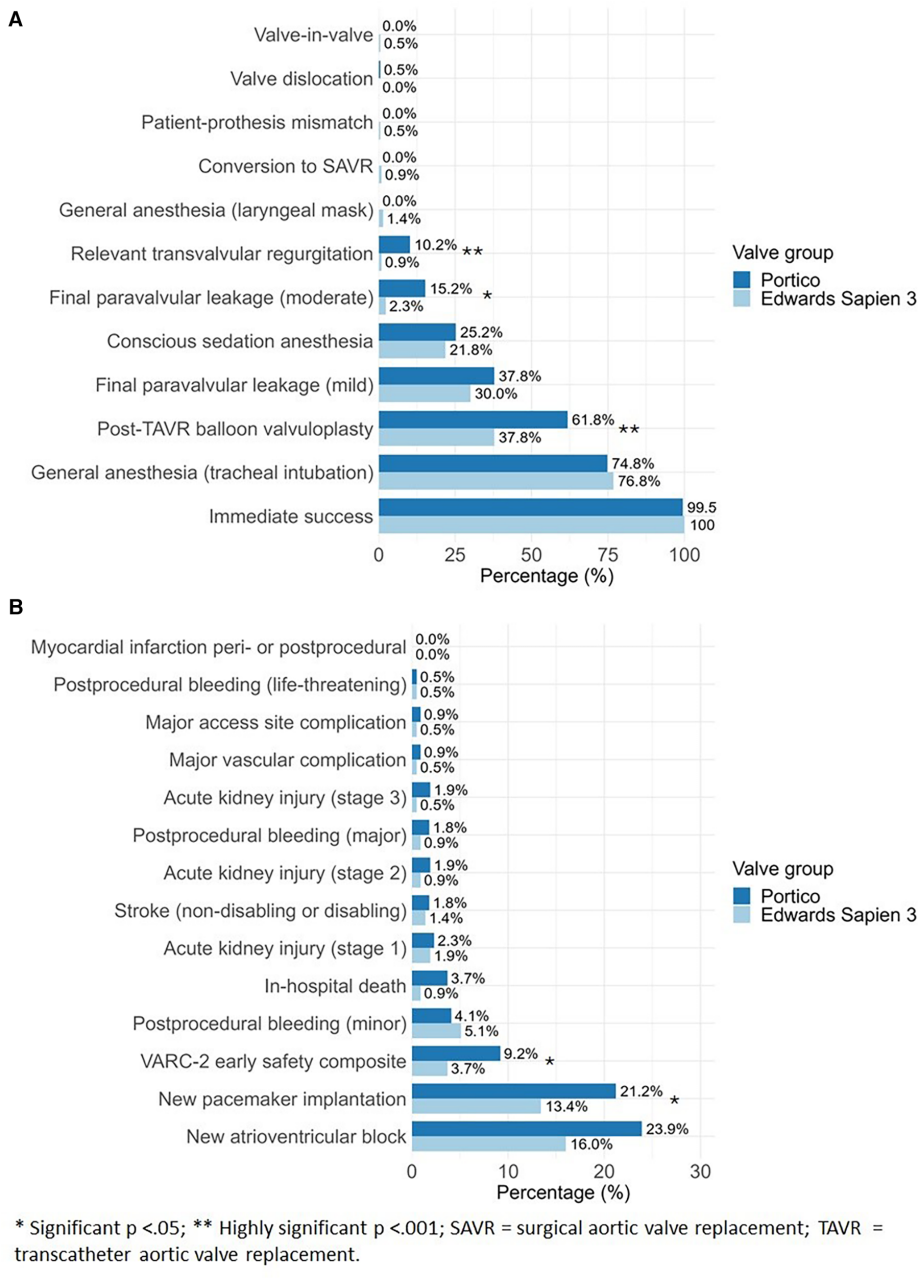
Comparative analysis between portico and Edwards Sapien 3 for (**A**) procedural details and (**B**) 30-days early clinical outcome after TAVR implantation.

Immediate post-interventional survival rate was 99.5% (*n* = 216) in Portico group and 100% (*n* = 217) in ES3 group (*p* = 1). There were significant differences between the Portico and ES3 groups in the usage of contrast medium, 127 (51.4) ml compared to 96.5 (46.0) ml (*p* < 0.001). Furthermore, the Portico group exhibited higher radiation exposure time [13.4 (5.96) min] and dose [44.3 (38.7) Gy·cm^2^] compared to the ES3 group with radiation exposure time of 12.9 (9.33) min and dose of 43.5 (63.4) Gy·cm^2^ (*p* = 0.002; *p* = 0.05), respectively. Lastly, balloon valvuloplasty occurred more frequently in the Portico group, with 134 cases (61.8%), compared to 82 cases (37.8%) in the ES3 group (*p* < 0.001). There were no significant differences in mild final paravalvular leakage (PVL) between Portico (37.8%, *n* = 82) and ES3 groups (30.0%, *n* = 65) (*p* = 0.105). However, there were significantly higher rates of moderate final PVL in the Portico group (15.2%, *n* = 33) compared to the ES3 group (2.3%, *n* = 5) (*p* < 0.001). Similarly, final relevant transvalvular regurgitation (TVR) was more frequently observed in the Portico group (10.2%, *n* = 22) compared to the ES3 group (0.9%, *n* = 2) (*p* < 0.001). In two cases, patients initially received the Portico valve; however, in one case, the valve was subsequently replaced with the ES3 valve due to high-grade insufficiency, while in the other case, the replacement was performed due to valve dislocation. Furthermore, in two other cases that received the ES3 valve, a conversion to surgical aortic valve replacement was necessary because of technical issues. Post-interventional echocardiography revealed patient-prosthesis mismatch in one patient of the ES3 group. Most valve implantations were performed under general anesthesia in both groups: 157 cases (74.8%) in the Portico group and 162 cases (76.8%) in the ES3 group (*p* = 0.712). Conscious sedation was used in 53 cases (25.2%) in the Portico group, compared to 46 cases (21.8%) in the ES3 group (*p* = 0.474). Details for procedural parameters are summarized in Table 2 and illustrated in Figure 2B.

**Table 2:** Procedural details.

		Portico	Edwards Sapien 3	*P*-value
		*N* = 217	*N* = 217	
Immediate success		216 (99.5%)	217 (100%)	1
Total procedural time (min)		82.7 (34.9)	80.9 (41.4)	0.043*
Anaesthetic procedure	Conscious sedation anesthesia	53 (25.2%)	46 (21.8%)	0.474
	General anesthesia (tracheal intubation)	157 (74.8%)	162 (76.8%)	0.712
	General anesthesia (laryngeal mask)	0 (0%)	3 (1.4%)	0.248
Contrast dye usage (ml)		127 (51.4)	96.5 (46.0)	<0.001**
Total radiation time (min)		13.4 (5.96)	12.9 (9.33)	0.002*
Radiation dose (Gy/cm^2^)		44.3 (38.7)	43.5 (63.4)	0.05
Patient-prothesis mismatch		0 (0%)	1 (0.5%)	1
Conversion to SAVR		0 (0%)	2 (0.9%)	0.476
Post-TAVR balloon valvuloplasty		134 (61.8%)	82 (37.8%)	<0.001**
Valve dislocation		1 (0.5%)	0 (0%)	1
Valve-in-valve		0 (0%)	1 (0.5%)	1
Final paravalvular leakage	Mild	82 (37.8%)	65 (30.0%)	0.105
	Moderate	33 (15.2%)	5 (2.3%)	<0.001**
Relevant transvalvular regurgitation		22 (10.2%)	2 (0.9%)	<0.001**
Total hospital length of stay (days)		8.00 [3.00, 70.0]	8.00 [2.00, 149]	0.672

Values are displayed as frequencies (percent), mean (standard deviation) and median [interquartile range]. SAVR, surgical aortic valve replacement; TAVR, transcatheter aortic valve replacement.

* Significant *p* < .05.

** Highly significant *p* < .001.

Among the ES3 group, the 29 mm valve size was the most frequently used, representing 39.2% (*n* = 85) of cases. The 29 mm valve size was also predominantly used for Portico valves, accounting for 40.6% (*n* = 88) of cases. The distribution of implanted prosthesis sizes is presented in Table 3.

**Table 3:** Implanted valve sizes.

	Portico	Edwards Sapien 3
	*N* = 217	*N* = 217
Valve size (mm)		
20	–	1 (0.5%)
23	16 (7.4%)	58 (26.7%)
25	48 (22.1%)	–
26	–	73 (33.6%)
27	65 (30.0%)	–
29	88 (40.6%)	85 (39.2%)

Values are displayed as frequencies (percent).

### Primary and secondary outcomes at 30-days

All clinical outcomes at 30-days are detailed in Table 4 and illustrated in Figure 2A. At 30-days, all-cause mortality was 3.7% (*n* = 8) in Portico group compared to 0.9% (*n* = 2) in ES3 group (*p* = 0.11). All reported deaths in the study occurred during the post-interventional hospital stay. Consequently, the in-hospital mortality rate can be considered equivalent to the 30-day mortality rate. There was a significantly higher occurrence of new PPM implantation in the Portico group compared to the ES3 group, with 21.2% (*n* = 46) and 13.4% (*n* = 29) respectively (*p* = 0.042). The VARC-2 early safety composite defining complications were observed significantly more often in Portico compared to ES3 group [9.2% (*n* = 20) vs. 3.7% (*n* = 8); *p* = 0.032]. There were no significant differences between the two groups in the occurrence of bleeding, vascular complications, major access site complication or acute kidney injury. No events of postinterventional myocardial infarction, ventricular perforation or valve-related dysfunction requiring repeat procedures were observed in either valve group.

**Table 4:** Early clinical outcomes (30-day).

		Portico	Edwards Sapien 3	*P*-value
		*N* = 217	*N* = 217	
Stroke (non-disabling or disabling)		4 (1.8%)	3 (1.4%)	1
Myocardial infarction peri- or postprocedural		0 (0%)	0 (0%)	NA
Postprocedural bleeding	Minor	9 (4.1%)	11 (5.1%)	0.833
	Major	4 (1.8%)	2 (0.9%)	
	Life-threatening	1 (0.5%)	1 (0.5%)	
Major vascular complication		2 (0.9%)	1 (0.5%)	1
Major access site complication		2 (0.9%)	1 (0.5%)	1
Acute kidney injury	Stage 1	5 (2.3%)	4 (1.9%)	0.77
	Stage 2	4 (1.9%)	2 (0.9%)	0.446
	Stage 3	4 (1.9%)	1 (0.5%)	0.368
New atrioventricular block		51 (23.9%)	34 (16.0%)	0.052
New pacemaker implantation		46 (21.2%)	29 (13.4%)	0.042*
In-hospital death		8 (3.7%)	2 (0.9%)	0.11
VARC-2 early safety composite		20 (9.2%)	8 (3.7%)	0.032*

Values are displayed as frequencies (percent), mean (standard deviation) and median [interquartile range].

* Significant *p* < .05.

### Risk predictors for new PPM implantation

**Figure 3:**
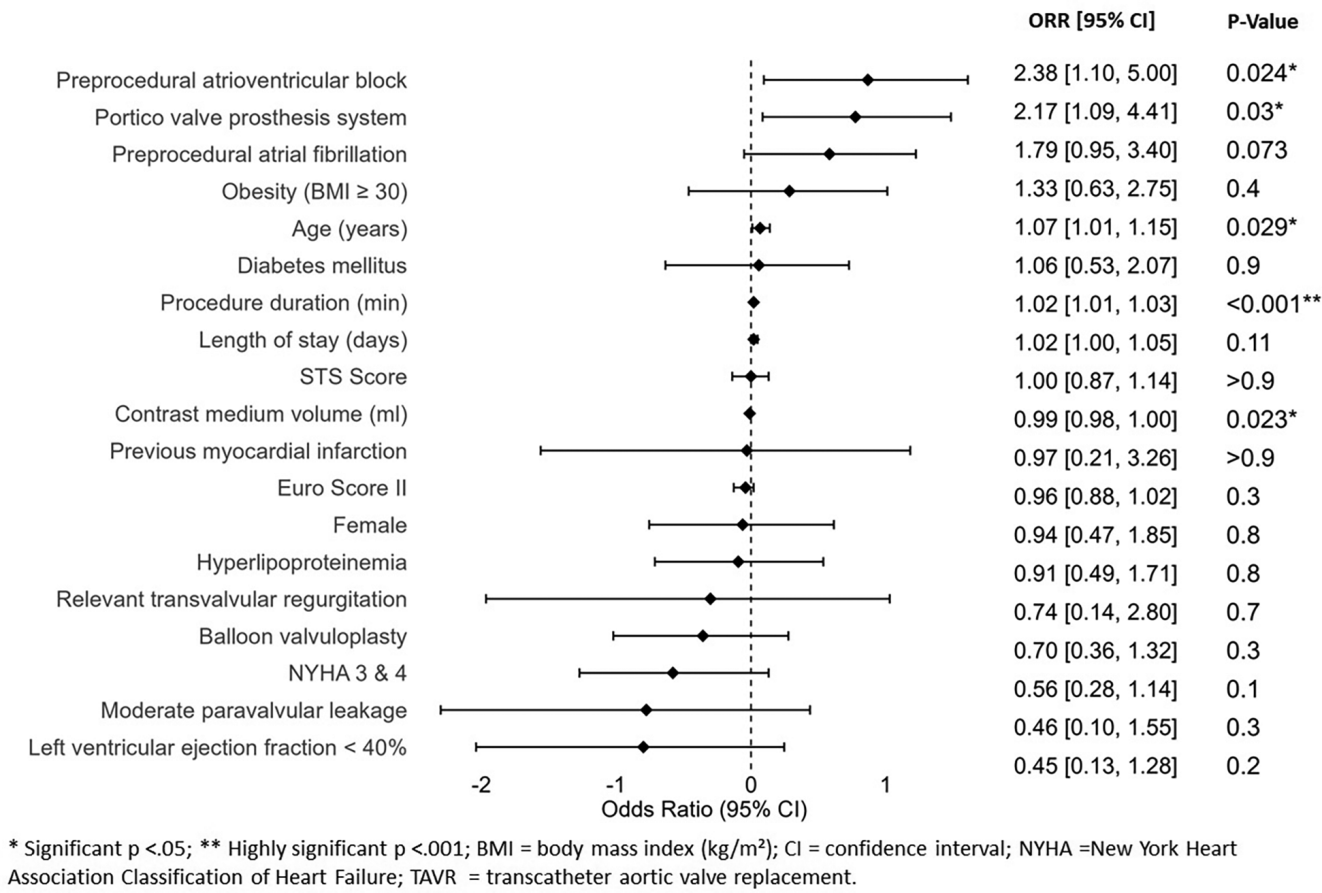
Risk factors associated with new pacemaker implantation post-TAVR.

In the multivariable logistic regression model several factors were identified as significant risk predictors for new PPM implantation (Figure 3). These included higher age (OR: 1.07; 95% CI: 1.01–1.15; *p* = 0.029), preprocedural AVB (OR: 2.38; 95% CI: 1.10–5.00; *p* = 0.024), the use of the Portico valve prosthesis (OR: 2.17; 95% CI: 1.09–4.41), and longer total duration of procedure (OR: 1.02; 95% CI: 1.01–1.03; *p* < 0.001).

## Discussion

In this comparative analysis involving Portico valve and ES3 valve, our findings revealed significant differences in the 30-day clinical outcomes. Summarizing, the key observations are as follows:

1. Patients with Portico valve showed higher rates of 30-day clinical complications. These included increased necessity for new PPM implantation and a greater overall 30-day safety risk as per the VARC-2 early safety composite endpoint.
2. Patients with Portico valve had a significantly higher usage of contrast dye, more radiation exposure, higher numbers of post-TAVR balloon-valvuloplasty and significantly higher rates of moderate PVL and aortic regurgitation compared to the ES3.
3. In our multivariable regression model the receival of Portico valve occurred as significant risk predictor for new PPM implantation alongside with advanced age, preexisting AVB and longer total procedure duration.

So far, there has been only one study that directly compared Portico and ES3 valve prostheses within a matched cohort consisting of 177 patients. In that study no significant differences in outcomes between the two valve prostheses were reported (11).

### Clinical outcomes

Immediate post-interventional success was high for both groups indicating a generally high level of safety for TAVR procedure. The 30-day mortality of 3.7% associated with Portico valve prostheses is comparable to earlier reported mortality rates in other trials (11–14). In contrast, the ES3 group showed a relatively low 30-day mortality rate of 0.9%, which aligns with previously reported data (15, 16). Considering the preprocedural calculated EuroScore II for each group, the expected mortality rates were anticipated to be quite similar. However, the observed mortality outcomes differed from these predictions, suggesting the presence of other factors that might have influenced the EuroScore II model.

New PPM implantation emerged as the second most frequent complication after new AVB. Our findings are consistent with previously reported data on new generation valve prostheses, demonstrating a relatively low overall rate of clinical complications except the occurrence of new PPM implantation (17). The rates of new PPM in patients with Portico valve prostheses were quite similar as observed by Mas-Peiro et al. (11). However, we found a significantly lower rate of PPM in patients receiving ES3 (21.2% for Portico vs. 13.4% for ES3). Previously reported rates of new PPM implantation with the ES3 valve show high fluctuation and a decreasing trend over the years. For instance, Murray et al. reported a rate of 25.5% (18), followed by De-Torres et al. reporting a rate of 19.1% in 2016 (19). Most recently, Monizzi et al. described a substantially lower rate of 6.3% in 2022 (16). The rate of 13.1% of PPM implantation in ES3 in our study aligns with the decreasing trend reported in previous studies. In our multivariable regression model, we found that the implantation of Portico valve prostheses remained an independent risk predictor for new PPM with twice the odds compared to patients receiving ES3 valve. These findings are consistent with previous studies, which show that PPM is more often associated with self-expandable prostheses than balloon-expandable prostheses (5, 6, 20).

In the context of VARC-2 early safety composite endpoint, we found a significantly higher rate in Portico valves compared to ES3 valves. Our findings suggest that patients receiving the Portico valve may be at a greater risk of experiencing one or more of these adverse events within the first 30-days following the procedure. This observation is in line with the findings from the prospective PORTICO IDE trial, which also reported higher event rates in the 30-day early composite endpoint for the Portico valve when compared to other commercially available valves (7).

### Procedural details

As for TAVR procedure significantly more contrast medium and radiation time was necessary for Portico valve prostheses. Despite the Portico valve group showing higher numbers in all AKIN stages, there were no significant differences observed in terms of postprocedural acute kidney injury when compared to ES3 group. However, the higher usage of contrast dye may be an important aspect when choosing a valve prosthesis for patients with preexisting kidney impairment.

While Mas-Peiro et al. did not report significant differences between the Portico and ES3 valve prostheses in terms of PVL, they did observe higher numbers of PVL in Portico valves (11). In our study, we also observed a higher occurrence of PVL and found a significant increase in moderate PVL and TVR in the Portico valve group. However, with the introduction and increasing usage of the Navitor valve prothesis, the latest iteration of Portico prothesis specifically designed to improve PVL outcomes, lower rates of PVL are likely to be observed in clinical practice. The higher rate of TVR in the Portico group may be attributed to the frequent need for post-dilation, which is sometimes required to ensure proper expansion of the prosthesis.

Notably, our study also affirmed the results reported by Mas-Peiro et al. regarding the favorable procedural outcomes associated with larger valve sizes.

## Limitations

The retrospective design of our study is a notable limitation as it relies on the analysis of existing medical records, introducing potential biases and limitations related to the collection and availability of data. Patients in our study were not randomly assigned to the treatment groups and although we attempted to address this limitation through propensity score matching, there is still the possibility of hidden confounders that may have introduced bias into our results.

The exclusion of a substantial portion of the patient cohort due to incomplete records introduces potential selection bias. This was a necessary step, as our study design required complete case analysis to facilitate appropriate patient matching. While this significantly reduced the patient sample size, it was essential for maintaining the methodological rigor of this comparative effectiveness research. Although this decision limits the generalizability of our findings, it enhances the validity of comparisons drawn from well-matched cohorts.

Although obtaining long-term outcomes would enrich our results, our study was specifically designed to provide real-world short-term outcome data between Portico and ES3 prostheses. The study, conducted with patients enrolled between 2018 and 2021, used VARC-2 criteria, the prevailing standard at the time. Although VARC-3 criteria were published in 2021, retrospective application was not feasible due to data constraints.

## Conclusion

In this propensity score matched analysis, we could observe significant higher rates of 30-day clinical complications as per VARC-2 criteria in Portico valve prostheses compared to ES3. Particularly, patients of advanced age and those with preprocedural kidney disease or significant rhythm disorders should be considered for balloon-expandable valve prostheses. The findings of this study highlight the importance of a personalized approach to valve selection in TAVR considering each patient's individual risk profile.

## Data Availability

The original contributions presented in the study are included in the article/Supplementary Material, further inquiries can be directed to the corresponding author.
